# Addition of Neuromuscular Electrical Stimulation, Based on the Level of Excitability, to an Intradialytic Twice‐Weekly Cycling Protocol: A Randomized Trial

**DOI:** 10.1002/pri.70143

**Published:** 2025-12-12

**Authors:** Beatriz da Costa Ferreira, Clara Narcisa Silva Almeida, Daniel da Costa Torres, Rafaela Regina de Souza Torres, João Luiz Quaglioti Durigan, Saul Rassy Carneiro, Laura Maria Tomazi Neves

**Affiliations:** ^1^ Faculty of Physiotherapy and Occupational Therapy Federal University of Para Belem Para Brazil; ^2^ Postgraduate Program in Human Movement Sciences Federal University of Para Belem Para Brazil; ^3^ Department of Physiotherapy Cosmopolitan Faculty Belem Para Brazil; ^4^ Laboratory of Muscle and Tendon Plasticity Graduate Program in Rehabilitation Sciences University of Brasilia Brasilia Brazil

**Keywords:** chronic kidney disease, intradyalitic, neuromuscular electrical stimulation

## Abstract

**Background and Purpose:**

Neuromuscular electrical stimulation (NMES) has shown promise as an adjunct or alternative to traditional training, but its optimal application in chronic kidney disease (CKD) population remains uncertain. This study aimed to compare the effects of the NMES intradialytic protocol combined with a traditional cycling on muscle strength and functional capacity in CKD patients.

**Methods:**

Fourteen patients were randomly assigned to either the experimental group (cycling plus NMES) or the control group (cycling only). Treatments were administered twice weekly for 8 weeks. Primary outcomes included muscle strength (isometric manual dynamometry) and functional capacity (1‐min sit‐stand test). Secondary outcomes included neuromuscular excitability, respiratory muscle strength, and quality of life.

**Results:**

After 8 weeks, repeated measures ANOVA showed no significant differences between the groups for any outcomes.

**Discussion:**

This study demonstrated that NMES was not superior to the cycling protocol in terms of peripheral muscle strength, functional capacity, neuromuscular excitability, respiratory muscle strength, and quality of life.

## 1 Introduction

Musculoskeletal dysfunction is a common and debilitating complication in individuals with chronic kidney disease (CKD), characterized by reduced muscle strength and mass, often referred to as uremic sarcopenia (Fahal 2014). This condition arises from a complex interplay of factors, including a decreased glomerular filtration rate (GFR), chronic inflammation, metabolic acidosis, reduced protein intake, insulin resistance, and hormonal imbalances. These factors activate proteolytic pathways and accelerate the loss of protein reserves (Mohanasundaram and Fernando 2022). Hemodialysis further exacerbates muscle impairment by causing the loss of amino acids and nutrients, which contributes to exercise intolerance, poor cardiorespiratory fitness, functional limitations, and increased mortality rates (Fahal 2014; Evans et al. 2022).

Physical exercise has been shown to mitigate some of these adverse effects in CKD. Aerobic and resistance training during hemodialysis sessions have been found to improve symptoms, enhance dialysis efficiency, increase bone mineral density (Liao et al. 2016), and improve quality of life (De Medeiros et al. 2017; Lin et al. 2021). Additionally, these interventions have been shown to enhance peak oxygen consumption and exercise capacity (Yabe et al. 2021), with higher adherence rates observed when exercise is performed during dialysis (Bernier‐Jean et al. 2022). Despite these benefits, muscle fatigue often limits the implementation of traditional exercise programs.

Neuromuscular electrical stimulation (NMES) has emerged as a promising alternative for enhancing muscle strength, endurance, functional capacity, and quality of life in individuals with chronic kidney disease (CKD) (Roxo et al. 2016; Valenzuela et al. 2020). NMES may benefit patients who are unable to engage in conventional exercise due to frailty or clinical limitations, as it can induce muscle contractions without active participation from the patient (Jang and Park 2021). Recent systematic reviews have highlighted the safety and feasibility of NMES, whether alone or combined with resistance or aerobic training, for this population (Valenzuela et al. 2020; Schardong et al. 2020). However, most studies have compared NMES with no intervention, and few have investigated whether combining NMES with aerobic exercise, such as cycle ergometry, provides additional benefits.

Electrolyte disturbances and altered neuromuscular excitability in CKD present challenges for the effective application of NMES (Barss et al. 2018; Dhondup and Qian 2017; Mahaldar 2012). These factors can impair motor unit recruitment and compromise the quality of evoked contractions, particularly in patients with disuse syndrome (Maffiuletti et al. 2018; Silva et al. 2018). Traditional NMES protocols often fail to optimize muscle recruitment in larger muscle groups and may exacerbate fatigue (Barss et al. 2018). To address these limitations, a chronaxie‐based NMES protocol has been proposed, utilizing electrodiagnostic tests to identify recruitment thresholds and determine the optimal pulse width for muscle contraction (Paternostro‐Sluga et al. 2002; Silva et al. 2018). This approach has been applied in critically ill patients with electrolyte imbalances, where it improves muscle activation despite the challenges presented by these conditions (Chleboun et al. 2007).

While the benefits of NMES and aerobic exercise are well‐documented individually, the potential synergy between a chronaxie‐based NMES protocol and cycling exercise in CKD patients remains unexplored. This knowledge gap is particularly relevant for clinical practice, as combining these interventions may enhance peripheral and respiratory muscle strength, functional capacity, and neuromuscular excitability. The aim of this study was to evaluate the effects of a chronaxie‐based NMES protocol combined with cycling exercise on peripheral and respiratory muscle strength, functional capacity, and neuromuscular excitability during intradialytic exercise. The hypothesis was that this combined approach would improve the quality of evoked contractions and optimize clinical outcomes in this high‐risk population.

## 2 Methods

### 2.1 Study Design

A randomized controlled, two‐arm, parallel, single‐blind clinical trial to evaluate the effects of a cycling protocol and chronaxie‐based NMES (intervention group) compared with a cycling protocol alone (control group) in intradialytic exercise. The study complies with the Consolidated Standards of Reporting Trials (CONSORT) statement for randomized trials (Cuschieri 2019). This research was carried out in accordance with the Declaration of Helsinki and the Nuremberg Code, respecting the Standards for Research Involving Human Beings of the Brazilian National Health Council (Resolution 466/12). The study was approved by the Research Ethics Committee of the Hospital das Clinicas Gaspar Vianna Foundation (Protocol n°. 5976962), and registered in the Brazilian Clinical Trials Registry (REBEC) in 01/04/2024, registration number: RBR‐8gj3fkt. All participants signed the informed consent form.

### 2.2 Participants

The study population consisted of individuals diagnosed with chronic kidney disease (CKD) who had been on hemodialysis for at least 3 months. The eligibility criteria included participants aged ≥ 18 years and with a functional level score of ≥ 4 (indicating the ability to transfer to a chair) on the Johns Hopkins Highest Level of Mobility Scale (Hoyer et al. 2016), ensuring that participants had sufficient mobility to safely engage in the prescribed exercise interventions. To minimize bias in assessing peripheral muscle strength and functional capacity, the study included sedentary individuals or those engaging in less than 300 min of moderate physical activity per week or performing strength training fewer than two times per week (Bull et al. 2020).

Participants were excluded if they had neuromuscular diseases, symptomatic ischemic heart disease, or a history of myocardial ischemia and angina within the previous 6 months, as these conditions could increase the risk of adverse cardiovascular events during aerobic exercise (Bernier‐Jean et al. 2022). Similarly, participants with hyperkalemia > 6 mmol/L or significant valvular heart disease were excluded to prevent potential complications that might arise during exercise interventions (Shibata and Uchida 2022; Bernier‐Jean et al. 2022). Participants with cognitive or functional impairments were also excluded to ensure accurate understanding and execution of the study procedures.

Due to the application of neuromuscular electrical stimulation (NMES), patients with implantable cardioverter defibrillators and epilepsy were excluded because of the risk of device interference or the potential to trigger seizures during current application. Peripheral edema and lower extremity ischemia were also excluded to prevent exacerbation of these conditions during NMES, and obesity (BMI > 35 kg/m^2^) was excluded to avoid alterations in electrical current impedance (Kamiya et al. 2016; Lago et al. 2022).

The researchers obtained medical history information through a review of medical records and interviews. The study was conducted at the Gaspar Vianna Clinical Hospital and the Monteiro Leite Hemodialysis Center in Brazil from July 2022 to May 2023.

### 2.3 Randomization

The randomization sequence was generated using the website www.random.org. An external investigator (LMTN), who was not involved in participant assessment or treatment, created the sequence and ensured allocation concealment by securely maintaining the list. Eligible volunteers were then randomly assigned using block randomization (two blocks of eight individuals) to either the control or intervention group by LMTN. A separate researcher (CNSA), blinded to group allocation, conducted participant evaluations before and after the intervention. Additionally, two other researchers (BCF and RRST) administered the intervention.

Due to the nature of the study, double blinding was not feasible. The intervention involved an additional therapy that was physically perceptible to participants, making it impossible to mask group allocation. Moreover, the researchers delivering the intervention had to actively engage with the specific therapeutic protocol, preventing them from being blinded. To minimize bias, outcome assessments were conducted by a separate blinded evaluator, and a standardized assessment protocol was followed.

### 2.4 Interventions

A supervised exercise program was implemented twice a week for 8 weeks, following the main protocol as outlined by Bündchen et al., until the second or third hour of dialysis, depending on the participant's clinical history of hypotension and hypoglycemia (Wilund et al. 2019). The intervention group followed a neuromuscular electrical stimulation (NMES) protocol based on chronaxie in combination with a cycling protocol, while the control group performed only the cycling protocol.

**FIGURE 1:**
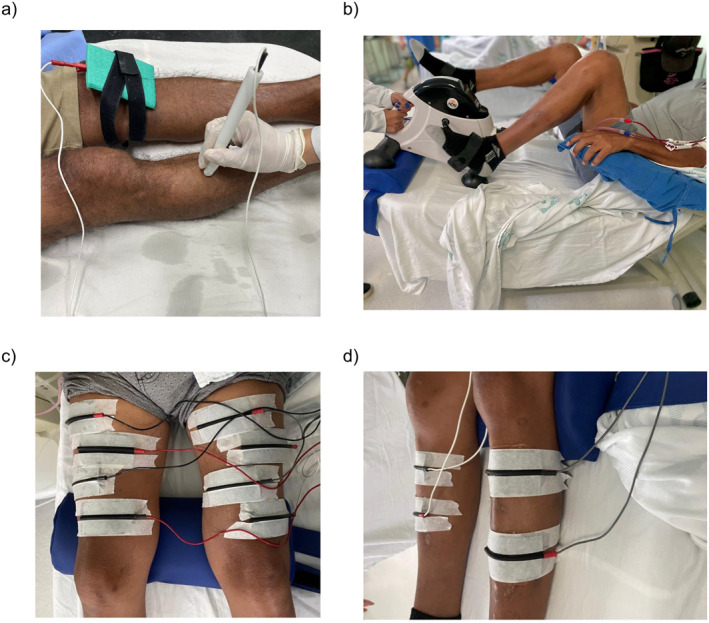
(a) Patient position and equipment for Stimulus electrodiagnosis test; (b) Patient positioning during cycling; (c) Electrodes position during NMES on vastus lateralis; (d) Electrodes position during NMES on anterior tibialis.

The cycling protocol for both groups consisted of exercise using a cycle ergometer in a supine position (Figure 1). The exercise time followed the following sequence: (1) 3 min of warm‐up; (2) active cycling with a starting time of 10 min, increasing by 5 min weekly until reaching 30 min, depending on the participant's tolerance; and (3) 3 min of cool‐down (Parker 2016). Exercise intensity was determined using heart rate reserve (Bündchen et al. 2021), with a target range between 40% and 70%, according to the heart rate reserve formulas (Yabe et al. 2021), and through a moderate to slightly difficult (Cuschieri 2019; De Araujo et al. 2020; Dhondup and Qian 2017; Fernandes et al. 2016) rating on the BORG perceived exertion scale (graded from 6 to 20) (Lambert et al. 2022; Crawford et al. 2018; Cabral et al. 2020).

After cycling, participants in the intervention group received neuromuscular electrical stimulation (NMES) bilaterally on the vastus lateralis (VL) muscle for 24 min (Valenzuela et al. 2020), followed by 24 min of NMES on the anterior tibialis (AT) muscle (Valenzuela et al. 2020), using a 4‐channel electrotherapy device (Figure 1). The primary muscle groups of the lower limb were prioritized, and NMES was applied at the motor points according to Botter et al. (2011). The parameters were individualized based on the assessment of neuromuscular excitability through the electrodiagnostic stimulus test, following the protocol proposed by Silva et al. (2018). Rheobase was initially assessed solely as a step to determine chronaxie, with the goal of identifying the threshold required to generate a muscle contraction. The baseline chronaxie evaluations for VL and AT were used to prescribe pulse width, starting from the smallest pulse width capable of eliciting a visible contraction. The protocol adopted for individual prescription involved using twice the value of the smallest pulse width.

NMES and stimulus electrodiagnosis were performed using the same device (Dualpex 071, Quark Medical, Piracicaba). NMES parameters were adjusted as follows: total time 24 min in each muscle, with pulse width twice the chronaxie, following the protocol proposed by Silva et al. (2018) This approach aimed to provide an individualized prescription and minimize potential neuromuscular disturbances associated with CKD (Paternostro‐Sluga et al. 2002; Russo et al. 2007; Pinheiro‐Dardis et al. 2017 Pinheiro‐Dardis et al. 2017).

Other parameters were adjusted based on key values from the latest reviews: pulse frequency of 65 Hz, ON/OFF time of 8/16 s, rise time of 2 s, and decay time of 2 s, totaling 60 contractions (Schardong et al. 2020; Valenzuela et al. 2020). The intensity of the current was fixed on the largest visible muscle contraction, according to the contraction quality scale proposed by Segers et al. aiming to achieve type 4 contractions (palpable and visible contraction with partial recruitment) in the first session and subsequently type 5 contractions (palpable and visible contraction with total recruitment), within the participant's maximum tolerance. After viewing the contraction initially evoked with the application of NMES, the patient was encouraged to perform a muscle contraction when receiving the stimulus.

### 2.5 Outcomes

#### 2.5.1 Primary Outcomes

Peripheral Muscle Strength: Knee extensors, knee flexors, and ankle plantar and dorsiflexors were assessed using a manual dynamometer, following established protocols (Mentiplay et al. 2015; Morin et al. 2023). Peak force data for the dominant limb were recorded in kgf. The dynamometer has good to excellent reliability for most isometric lower limb strength measurements, particularly for proximal muscles (hip and knee ICCs ≥ 0.70; ankle ICCs = 0.31–0.79) (Mentiplay et al. 2015). Tests were conducted in a sitting position for knee muscles and supine for ankle muscles, with each participant performing a four‐second maximum voluntary isometric contraction under consistent examiner supervision.

#### 2.5.2 Secondary Outcomes

Functional Capacity: The 1‐min sit‐stand test (STS1) was performed to assess functional capacity and lower limb endurance (Crisafulli and Clini 2010). Participants completed the maximum number of repetitions of sitting down and standing up from a chair (46 cm in height, without armrests) without using their upper limbs, within 1 min. They began the test seated, with arms crossed, placing each hand on the contralateral shoulder. A cycle of sitting and standing was counted as one repetition. STS1 is a reliable method (Bohannon and Crouch 2019; Segura‐Ortí and Martínez‐Olmos 2011) and is also correlated with quadriceps thickness in individuals with chronic kidney disease (CKD) in the pre‐dialysis stage (Costa et al. 2022).

Neuromuscular Excitability: The stimulus electrodiagnosis test was used to evaluate the neuromuscular excitability of the vastus lateralis (VL) and anterior tibialis (AT) muscles to obtain the rheobase and chronaxie variables (Paternostro‐Sluga et al. 2002). Rheobase refers to the minimum current intensity required to produce a slight muscular contraction (i.e., reaching the neuromuscular excitability threshold), while chronaxie, which is determined after rheobase, is defined as the shortest pulse duration needed to reach the neuromuscular excitability threshold with a square pulse current at twice the intensity of the rheobase (Fernandes et al. 2016).

During the test, patients remained semi‐recumbent with extended limbs. The skin was cleaned, and trichotomy was performed if needed. A reference electrode (anode) was placed on the contralateral patella. Motor points were identified using a pen‐shaped cathode electrode to locate the lowest current required for muscle contraction (Figure 1) (Roxo et al. 2016; Paternostro‐Sluga et al. 2002; Botter et al. 2011). Rheobase and chronaxie were measured using a biphasic rectangular current with a 25 cm^2^ silicone carbon cathode electrode (De Araujo et al. 2020).

For measuring rheobase, the intensity was increased from 1 to 69 mA with individual increments of 1 mA until causing a slight and visible muscle contraction. For measuring chronaxie, the pulse duration was gradually increased from 20 μs to 1 ms until a light and visible muscle contraction was provoked (Paternostro‐Sluga et al. 2002). Average chronaxie values are approximately 110 microseconds, with values ≥ 1000 microseconds characterizing the presence of neuromuscular electrophysiological disorder (Paternostro‐Sluga et al. 2002).

Respiratory muscle strength: MIP and MEP were measured using an analog manovacuometer (−120 to +120 cmH_2_O) at 4 cmH_2_O intervals. Participants sat with knees and hips at 90°, nose occluded, and received verbal feedback. MIP was measured from residual volume and MEP from total lung capacity, following Brazilian guidelines (Souza 2002). Predicted values were based on Sanchez et al.'s BMI‐based reference equation (Sanchez et al. 2018).

Quality of life (QoL): QoL was assessed using the WHOQOL‐bref, a widely used generic questionnaire for both healthy and sick populations (Fleck et al. 2000; Kluthcovsky and Kluthcovsky 2009). It includes 26 items covering four domains—physical, psychological, social relationships, and environment—plus general QoL and health satisfaction. Each item is rated on a one to five Likert scale, with higher scores indicating better QoL (Kluthcovsky and Kluthcovsky 2009).

### 2.6 Data Analysis

The sample size was calculated using isometric muscle strength of the knee extensors as the primary outcome using G*Power software, according to the study conducted by Suzuki et al. (2018). We estimated an effect size of 1.8 in the difference between means and standard deviation in quadriceps isometric muscle strength after 8 weeks of treatment. Considering a study power of 80%, a significance level of 95%, and a sample size ratio of 1:1 (control group or intervention group), we reached the estimated number of 6 subjects per group.

The normality of the continuous data was analyzed using the Shapiro‐Wilk test. Variables with parametric distribution are expressed as mean ± standard deviation, while variables with non‐parametric distribution are expressed as median and interquartile range 25%–75%.

Comparisons between groups of categorical variables were performed using the chi‐square or Fisher's exact test, for continuous variables, the Student's *t*‐test was used for variables with parametric distribution, and the Mann‐Whitney test for variables with non‐parametric distribution. ANOVA for repeated measurements was applied for group‐time interactions. An intention‐to‐ treat analysis was performed for all randomized participants. For all statistical analyses, we used the Jamovi software version 2.3.13.

## 3 Results

### 3.1 Flow of Participants

**FIGURE 2:**
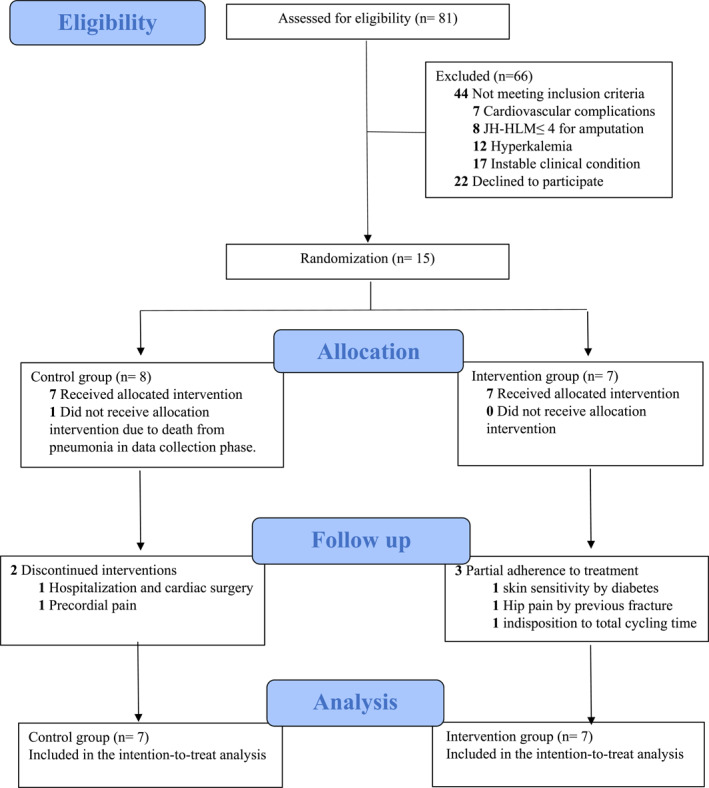
Flow diagram of patient recruitment.

The eligibility of 81 patients was assessed, of whom 37 met all inclusion criteria, but only 15 agreed to participate in the study and were included and randomly allocated to the control group (*n* = 8) or intervention group (*n* = 7). One individual from the control group died of pneumonia during the data collection phase, and therefore, 14 participants were included for data analysis (Figure 2). Clinical and sociodemographic data are presented in Table 1. No differences were found regarding sample characteristics.

**TABLE 1:** Characteristics of participants at baseline.

	Control	Intervention	*p*‐value
Age (Years)	47 ± 19.3	54.3 ± 7.4	0.3
Sex			
Female	3 (21.4)	2 (14.3)	1.0
Male	4 (28.6)	5 (35.7)	
Education			
No schooling or incomplete elementary education	5 (35.7)	4 (28.6)	1.0
Incomplete high school	1 (7.1)	1 (7.1)	
Complete high school	1 (7.1)	2 (14.3)	
Marital status			
Single	4 (28.6)	2 (14.3)	0.2
Married/Common‐law union	2 (14.3)	4 (28.6)	
Divorced	1 (7.1)	1 (7.1)	
Occupation			
Employed	0	1 (7.1)	1.0
Retired	7 (50)	6 (42.9)	
Habitation zone			
Urban	3 (21.4)	6 (42.9)	0.2
Rural	4 (28.6)	1 (7.1)	

Clinical characteristics			
BMI (kg/m^2^)	24.6 ± 7	22.9 ± 4.1	0.5
Functional assessment			
JHLM ≥ 4 ≤ 8	0	2 (14.3)	0.4
JHLM = 8	7 (50)	5 (35.7)	
Physical activity routine			
Sedentary	6 (42.9)	7 (50)	
2 times a week	1 (7.1)	0	1.0
% VL chronaxie ≥ 1000 μs	3 (42.8)	2 (28.6)	
% AT chronaxie ≥ 1000 μs	3 (42.8)	1 (14.3)	
Cause of CKD			
Diabetes mellitus	1 (7.1)	0	0.7
Arterial hypertension	1 (7.1)	1 (7.1)	
Diabetes and hypertension	5 (35.7)	4 (28.6)	
Other	0	2 (14.3)	
Dialysis time (years)	7 [2.5–11.5]	1.7 [1.2–7.5]	0.4

Laboratory tests			
Urea (mg/dL)	121 ± 35.2	132.6 ± 35.7	0.5
Creatinine (mg/dL)	8.9 ± 3.9	9.2 ± 5.5	0.9
Kt/V	1.5 ± 0.1	1.2 ± 0.1	0.06
GFR	6.1 ± 1.5	9.4 ± 6	0.2
Potassium (mEq/L)	5.1 ± 0.8	5.6 ± 0.4	0.1
Sodium (mEq/L)	133.1 ± 4	133.8 ± 4,1	0.7
Hemoglobin (g/dL)	11 [10.4–11.9]	10.8 [10.4–11]	0.6

*Note:* Data expressed as *n* (%), mean ± standard deviation or median [25%–75%].

Abbreviations: BMI, body mass index; JH‐HLM, Johns Hopkins highest level of mobility (Scale: Level 4‐transfer to armchair, Level 8‐ambulation ≥ 75 m); CKD, chronic kidney disease; GFR: glomerular filtration rate; Kt/V, dialysis adequacy index.

### 3.2 Effects of the NMES Protocol

Repeated measures ANOVA comparing treatment effects in group‐time interactions showed no statistically significant differences between groups for Peripheral Muscle Strength (Dorsiflexors *p* = 0.5; Plantar flexors *p* = 0.7; Knee Extensors *p* = 0.8; Knee Flexors *p* = 0.2); Functional Capacity *p* = 1.0; Respiratory Muscle Strength (MIP *p* = 0.4; MEP: *p* = 0.1); Neuromuscular excitability (VL Chronaxie *p* = 0.1; VL Rheobase *p* = 0.08; TA Chronaxie *p* = 0.7; TA Rheobase *p* = 0.5); Quality of Life (Physical *p* = 0.2; Psychological *p* = 0.2; Social *p* = 0.4; Environmental *p* = 0.7; Self‐assessed *p* = 1.0; General QoL *p* = 0.6). Although the intervention group demonstrated positive changes in primary and secondary outcomes, the results were not statistically significant (Table 2). Thus, the analysis found insufficient evidence to support the benefits of adding NMES to a cycling protocol.

**TABLE 2:** Description of group performance with repeated ANOVA measurements for group‐time interactions.

	Week 0		Week 4		Group‐time interaction		
	Control	Intervention	Control	Intervention	F	*p*‐value	*η* ^2^p
Peripheral muscle strength							
Dorsiflexors (kgf)	8.6 ± 2.8	9.6 ± 2.4	8.6 ± 2.9	10.6 ± 2.6	0.374	0.552	0.030
Plantar flexors (kgf)	8.2 ± 2	9.5 ± 1.7	9.3 ± 1.3	10.4 ± 2	0.0901	0.769	0.007
Knee extensors (kgf)	11.9 ± 3	13.6 ± 4.4	11.1 ± 2.9	12.4 ± 1.8	0.0596	0.811	0.005
Knee flexors (kgf)	8.9 ± 3.2	13.2 ± 4.9	9.8 ± 3.6	12.2 ± 3.6	1.5715	0.234	0.116
Functional capacity							
STS1 (repetitions)	16.7 ± 10.8	15.1 ± 8.9	18.2 ± 9.6	16.7 ± 10.6	0.000	1.0	0.000
Respiratory muscle strength							
MIP (cmH2O)	60 [38–96]	40 [40–58]	70.8 ± 37.9	65.1 ± 32.2	0.742	0.406	00.58
% of predicted	58 [34–84.5]	40 [37.5–61.5]	65 ± 35.8	64 ± 26.7	1.08	0.319	0.083
MEP (cmH2O)	72.5 ± 34.1	56.2 ± 24.2	72 ± 33.7	66.2 ± 24.7	2.09	0.174	0.148
% of predicted	74.4 ± 39.1	58.5 ± 24.5	73.7 ± 38.2	68.7 ± 22.8	2.19	0.164	0.154
Neuromuscular excitability							
VL‐chronaxie (μs)	700 [450–1000]	400 [250–700]	541.6 ± 287	542.8 ± 331	2.75	0.123	0.18
TA‐chronaxie (μs)	500 [325–900]	400 [400–600]	700 [500–700]	400 [300–750]	0.3373	0.572	0.27
VL‐rheobase (mA)	7.2 ± 2.3	8.3 ± 1.9	8.7 ± 2.7	9.2 ± 2.3	0.321	0.0860	0.774
TA‐rheobase (mA)	9.5 ± 3.9	9.7 ± 1.9	9.8 ± 2.4	9.2 ± 2.1	0.4360	0.522	0.035
Quality of life							
Physical domain	10.1 ± 2.8	11.4 ± 2.7	11.9 ± 3.2	10.8 ± 3.1	1.450	0.252	0.108
Psychological domain	13.7 ± 1.1	13.9 ± 1.6	13.7 ± 1.8	14.9 ± 1.4	1.37	0.265	0.102
Social domain	13.5 ± 2.5	13.9 ± 3.4	13.1 ± 3.6	11.9 ± 2.7	0.692	0.422	0.055
Environmental domain	12 ± 2.6	13 ± 1.5	13.9 ± 2.2	14.5 ± 1	0.149	0.706	0.012
QoL self‐assessed	14 ± 2	14.6 ± 3.2	11.6 ± 1.5	12.6 ± 3.7	0.000	1.0	0.000
General QoL	12.3 ± 2	12.9 ± 1.9	13 ± 1.1	13.1 ± 1.1	0.195	0.667	0.016

*Note:* Data expressed as mean ± standard deviation, median [25%–75%].

Abbreviations: cmH2O, centimeters of water; μs, microseconds; kgf, kilogram‐force; mA, milliamperes; MEP, maximum expiratory pressure; MIP, maximum inspiratory pressure; QoL, Quality of life; TA, tibialis anterior; VL, vastus lateralis; STS 1, 1‐min sit and stand test.

### 3.3 Compliance With the Trial Method

Of the seven participants in the control group, two patients interrupted the protocol, one due to recurrent episodes of hypotension from dialysis, and the other due to elective cardiac surgery, completing only 37.5% of sessions (6 of 16). Of the seven participants in the experimental group, three completed all 16 prescribed sessions but adhered partially to the protocol, adapting the total time of EENM or cycling. One participant completed 25% of the full protocol session due to indisposition during cycling, and the other two performed 37.5%, due to skin sensitivity by diabetes or hip pain by previous fracture. These patients were included in the intention to treat analysis.

The average current intensity used in NMES was 16.3 ± 7.1 mA for the vastus lateralis and 19.1 ± 7.6 mA for the tibialis anterior, respecting the tolerance of participants. These parameters generated type 4 contractions, according to the contraction quality scale proposed by Segers et al. The perceived exertion on the BORG scale during cycling remained between “mild” and “slightly intense” for both groups. Table 3 presents the other intervention monitoring variables.

**TABLE 3:** Intervention monitoring data.

	Control	Intervention
Active cycling time (min)	25 [20–30]	20 [15–27.5]
HR reached (bpm)	101.7 ± 21.5	89.2 ± 16.7
% of HR achieved	14 [9–25]	19 [8–32.5]
Perceived exertion (BORG 6–20)	12 [11–12]	12 [12–12.5]
Repetitions per minute in cycling	58.8 ± 17.1	67.6 ± 16.7
Current intensity in VL (mA)	N/A	16.2 ± 7
Quality of contraction in VL^a^	N/A	4.2 ± 0.7
Current intensity in TA (mA)	N/A	19.1 ± 7.6
Quality of contraction in TA^a^	N/A	4 ± 0.8

Abbreviations: bpm, beats per minute; HR, Heart rate; mA, milliampere;.Min, minutes; N/A, not applicable; TA, tibialis anterior; VL, vastus lateralis.

^a^ Contraction quality scale proposed by Segers et al. 22 Level 4‐ palpable and visible contraction (partial muscle recruitment); data expressed as mean ± standard deviation or median [25%–75%].

## 4 Discussion

The present study found no significant differences in functional capacity, peripheral and respiratory muscle strength, neuromuscular excitability, or quality of life between the control and intervention groups. These findings contrast with previous studies that demonstrated increased quadriceps strength in exercise programs using neuromuscular electrical stimulation (NMES) lasting from five to 20 weeks (Schardong et al. 2020; Bündchen et al. 2021). The combination of interventions in our study may have been limited by participants' low tolerance to the protocol, potentially restricting gains in the intervention group.

Individual factors may influence the response to NMES. Segers et al. (2014) identified responder and non‐responder patients, linking poor response to factors such as vasopressors, edema, and sepsis. In this study, some participants had chronaxie values ≥ 1000 μs (Table 1), suggesting neuromuscular electrophysiological disorders consistent with denervation (Paternostro‐Sluga et al. 2002; Russo et al. 2007). Denervation affects neuromuscular excitability, which can also be altered by hyperkalemia. Our patients had elevated serum K+ levels, likely due to uremic depolarization (Krishnan et al. 2005). Since neuromuscular excitability in CKD is underexplored, these factors may explain the differing results compared to recent reviews and trials.

According to the meta‐analysis by Schardong et al. (2020), NMES promoted increased lower limb strength and functional capacity, with a large effect size, regardless of whether it was combined with other therapies. However, our study found the opposite result. This discrepancy may be explained by limitations in the protocol. Despite individualized prescriptions, the current intensity was lower than in other studies due to participant tolerance or neuromuscular excitability abnormalities in chronic kidney disease (CKD) (Valenzuela et al. 2020).

We achieved partial muscle fiber recruitment (type 4, according to Segers et al. 2014), which is considered effective but not ideal. Other authors recommend sustained, tetanic contractions with visible or palpable superior patellar glide (Maffiuletti et al. 2018), which we did not achieve due to the participants' limited tolerance to the current. Moraes et al. (2022) addressed this issue by incorporating a warm‐up period, starting at 20% of the previous session's intensity and increasing by 20% per minute until the fifth minute. In our study, we only included an adaptation period during the first session. Incorporating a warm‐up and cool‐down period in each session may improve protocol adherence and participant tolerance.

The adequacy of NMES dosage in CKD patients remains uncertain, as previous studies did not assess contraction quality, tolerance, or adverse events (Schardong et al. 2020). Given that contraction intensity and treatment duration are crucial for meaningful muscular adaptations, their effectiveness is particularly relevant for individuals at risk of sarcopenia. Additionally, factors such as a sedentary lifestyle, aging, and CKD etiology create barriers to exercise (Gungor et al. 2021), further contributing to muscle weakness and reduced functional capacity (Müller‐Ortiz and et al. 2019). Despite these challenges, most NMES studies have reported positive effects, including improved six‐minute walk test performance (Liao et al. 2016; Ferrari et al. 2020). Similarly, CKD patients demonstrated enhanced performance in STS1 assessments, regardless of the intradialytic exercise modality used (Bogataj et al. 2019). These benefits may be attributed to the unique physiological effects of NMES, which synchronously recruits both slow and fast muscle fibers, promoting strength and endurance gains—an advantage particularly relevant in cases of severe muscle impairment (Maffiuletti et al. 2018; Silva et al. 2018).

To date, the prescription of NMES parameters based on neuromuscular excitability has not been reported for the CKD population. According to Maffiuletti et al. (2018), assessing the magnitude of electrically evoked contractions is essential for ensuring treatment effectiveness and is the only valid method for prescribing NMES. The study findings indicate that decreased neuromuscular excitability is associated with elevated chronaxie values, which require greater pulse widths to achieve strong contractions (Pieber et al. 2015). Additionally, patient responses to NMES may be influenced by the level of clinical impairment (Maffiuletti et al. 2018; Segers et al. 2014; Pieber et al. 2015). However, our study did not observe significant changes in neuromuscular excitability between groups. Given the lack of baseline alterations in excitability, it is possible that stimulation based on chronaxie had a minimal effect in our study.

This study found no differences in quality of life between groups. While a CKD‐specific assessment tool exists (Hussien et al. 2021), general questionnaires also reliably measure health‐related quality of life (Lin et al. 2021; Suzuki et al. 2018). Improvements in physical domains are typically linked to increased strength, functional capacity, and hemodialysis recovery time (Bündchen et al. 2021), but these effects were not observed in this trial. Despite NMES being widely used in clinical practice, the study suggests that twice‐weekly NMES during intradialytic sessions, based on excitability levels, does not provide significant long‐term benefits over traditional cycling for CKD patients.

This study has limitations. When assessing the main factors affecting exercise tolerance in this population, it is important to consider the impact these factors have on full adherence to the protocol. Additionally, the side effects of dialysis must be considered, as muscle fatigue is the primary barrier to exercise for individuals with CKD, followed by their overall clinical condition and associated comorbidities (Hannan and Bronas 2017). Twelve of the 16 participants experienced episodes of hypotension, hypoglycemia, fatigue, cramps, and hypertensive spikes because of dialysis sessions. On average, symptoms were present in 19.6% of sessions in the control group and 28.3% in the intervention group. These factors could potentially influence both the progression of the exercise protocol and the study outcomes.

The use of NMES with extended legs was a limiting factor, as it conflicted with the muscle length‐tension relationship, potentially affecting muscle strength gains. However, this position was chosen due to the hemodialysis chair available at the research center. Another limitation of the protocol was the absence of FES‐cycling. This study employed a frequency of 65 Hz, which, although considered high, aligns with the average used in NMES studies (Valenzuela et al. 2020; Schardong et al. 2020). FES‐cycling alone is associated with a higher incidence of fatigue (Ibitoye et al. 2016), and when combined with the high frequency used in this study, it may have further limited adherence to the full protocol, particularly given the fragility of participants with chronic kidney disease.

We suggest that in future studies the use of NMES should consider strategies to delay the onset of muscle fatigue, including the optimization of electrode positioning and modification of patterns of stimulation and its parameters to the CKD population. Finally, weekly training with a frequency equal to or greater than three times, associated with a longer follow‐up period and periodic reassessments during treatment could better elucidate the responses to intradialytic exercise.

## 5 Implications to Physiotherapy in Practice

This study suggests that while the addition of neuromuscular electrical stimulation (NMES) did not provide superior benefits compared to an isolated cycling protocol in terms of peripheral muscle strength, functional capacity, neuromuscular excitability, respiratory muscle strength, or quality of life for individuals with chronic kidney disease undergoing hemodialysis, it underscores the potential value of an individualized NMES prescription. By tailoring stimulation based on neuromuscular excitability, future studies could yield more effective interventions for this population, offering deeper insights into how exercise and neuromuscular modulation can be optimized for improved outcomes.

## Author Contributions

Clara Narcisa Silva Almeida and Beatriz da Costa Ferreira conceived and designed the study. Beatriz da Costa Ferreira and Rafaela Regina de Souza Torres conducted the experimental procedures. Daniel da Costa Torres, Laura Maria Tomazi Neves, and João Luiz Quaglioti Durigan contributed to the interpretation of the findings. Saul Rassy Carneiro performed the statistical analyses and contributed to data interpretation. Beatriz da Costa Ferreira and Rafaela Regina de Souza Torres led the drafting of the manuscript. All authors critically reviewed the content, contributed to the refinement of the research, and approved the final version of the manuscript.

## Funding

This work was supported by Federal University of Para through the Institutional Scientific Initiation Scholarship Program (Grant No: 23768.009471/2020‐69)

## Disclosure

The authors have nothing to report.

## Ethics Statement

This research was carried out in accordance with the Declaration of Helsinki and the Nuremberg Code, respecting the Standards for Research Involving Human Beings of the Brazilian National Health Council (Resolution 466/12). The study was approved by the Research Ethics Committee of the Hospital das Clinicas Gaspar Vianna Foundation (Protocol n°. 5976962), and registered in the Brazilian Clinical Trials Registry (REBEC) on 01/04/2024, registration number: RBR‐8gj3fkt. All participants signed the informed consent form.

## Consent

Written informed consent was obtained from all participants prior to their inclusion in the study.

## Conflicts of Interest

The authors declare no conflicts of interest.

## Data Availability

The datasets generated and analyzed during the current study are available from the corresponding author (Laura Maria Tomazi Neves) on reasonable request.
